# Viral vector production in the integrity^® ^iCELLis^® ^single-use fixed-bed bioreactor, from bench-scale to industrial scale

**DOI:** 10.1186/1753-6561-7-S6-P59

**Published:** 2013-12-04

**Authors:** Alexandre Lennaertz, Shane Knowles, Jean-Christophe Drugmand, Jose Castillo

**Affiliations:** 1 ATMI LifeSciences, Brussels, 1120, Belgium

## Introduction

Wild-type or recombinant viruses used as vaccines and human gene therapy vectors are an important development tool in modern medicine. Some have demonstrated high potential such as lentivirus, paramyxovirus and adeno-associated-virus (AAV). These vectors are produced in adherent and suspension cell cultures (e.g. HEK293T, A549, VERO, PER.C6, Sf9) using either transient transfection (e.g PEI, calcium phosphate precipitation) or infection (e.g. modified or recombinant viruses) strategies. Most of these processes are currently achieved in static mode on 2-D systems (Roller Bottles, Cell Factories, etc.) or on suspended microcarriers (porous or non-porous). However, these two systems are time-consuming (large numbers of manipulation, preparation of equipment, etc.) and hardly scalable. In regards to process simplification and traceability, Integrity^® ^iCELLis^® ^bioreactors offer a new solution for scalability and monitoring of adherent cell cultures.

### The Integrity iCELLis Bioreactor

Integrity^® ^iCELLis^® ^bioreactors from ATMI LifeSciences were designed for adherent cell culture applications such as recombinant protein, viral vaccine and gene therapy vector production. Using PET carriers trapped into a fixed-bed, cells grow in a 3-D environment with temperature, pH and dissolved oxygen controls. The iCELLis technology can be used at small-scale (the iCELLis nano from 0.5 to 4 m^2^) and manufacturing scale (iCELLis 500 from from 66 to 500 m^2^) which eases process scale-up and its overall utilization.

## Materials and methods

All the experiments described here have been performed in the bench-scale and pilot scale iCELLis bioreactors containing iPack carriers made of 100% pure non-woven PET fibers. Crystal violet was used for cell nuclei counts from carriers.

### Recombinant viral vectors production

Some recombinant entities are produced in the iCELLis bioreactors using hybrid vectors. For example, A549-stable packaging cell line, maintained in Optipro medium + 1% FBS, can deliver recombinant AAV vectors frequently used in gene transfer applications (Inserm UMR 649, Institut de Recherche Thérapeutique).

Alternatively, other rAAV vectors are obtained by transient transfection. In this case, HEK293-T cells are regularly found to be sensitive to the viral DNA and transfection reagent complex (generally polyethylenimine - PEI or phosphate calcium precipitate). The transfer of the transfection process from static or dynamic systems to the iCELLis bioreactors requires some adaptation in order to fully benefit of both technologies. Using a fluorescent protein marker, the transfected cells can be observed during the culture and the viral vectors can be quantified after the harvest.

Transfection method using the PEI/DNA complexes is frequently found in cell suspension processes due to its high efficiency and adaptability to high-throughput systems. The circulation pattern of the medium through the fixed-bed of the iCELLis system allows a good contact between cells and transfection complexes.

The transfection by phosphate precipitation is a static method where the DNA precipitates settle on the cells. For this reason, it is difficult to apply this technic in dynamic conditions. To be able to implement it in the iCELLis bioreactor, the agitation speed has to be minimal to get a medium circulation through the fixed-bed. This maintains the precipitate in suspension while giving the longest contact time between these precipitates and the cells. The iCELLis system with its pH regulation and low-shear circulation is well adapted for this method sensitive to small pH changes and reagent mix.

## Results

### Recombinant adeno-associated virus vector production

Recombinant AAV vectors were produced in an A549 based stable packaging cell line containing the AAV2 rep and cap genes from various AAV serotypes. Using a dual adenovirus infection (wild-type Ad5 followed by hybrid Ad/AAV) in the iCELLis nano bioreactor under perfusion mode, recombinant particles were harvested up to 96 hours post-infection. The expression levels of the AAV2 rep and cap genes from various AAV serotypes were assessed by western-blot and qPCR. This 8-days process demonstrated higher vector particles production in the iCELLis bioreactor compared to CS-5 control (4.5 × 10^8 ^*vs *3.1 × 10^8 ^vg/cm^2^, 72 h after the first infection) (Inserm UMR649, Institut de Recherche Thérapeutique).

**Figure 1:**
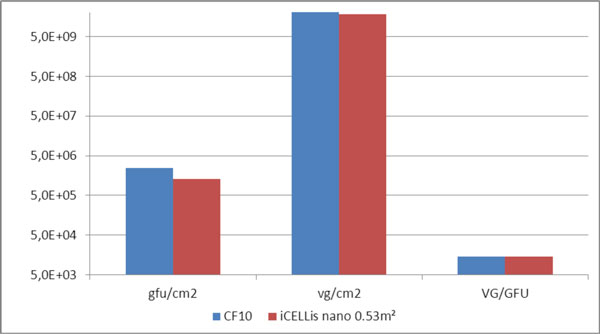
**Comparison of Green Fluorescent Units and Viral Genome/cm^2 ^and VG/GFYU ratio in the CS10 and iCELLis nano 0.53 m^2^**.

Triple transient transfection using PEI was performed in the iCELLis nano system (0.53 m^2^, 40 mL fixed-bed) for the production of serotype 5 AAV in HEK 293T cells. Cells were seeded at 80,000 cells/cm^2 ^in the CS10 and the iCELLis bioreactor. Twenty-four hours post-inoculation, the DNA-PEI mix containing the GFP gene was added to fresh medium inside the bioreactor. Cells were still growing on the carriers after the transfection. The expression of GFP by cells demonstrated that the transfection had a high efficiency rate in both vessels (FACS analysis on sampled carriers for the iCELLis bioreactor). Green Fluorescent Units (GFU) and Viral Genome (VG) were measured for the CS10 control and the iCELLis nano bioreactor. Viral particles were harvested using a freeze/thaw method, suboptimal in the case of the iCELLis system. The GFU and VG titers/cm^2 ^in the iCELLis bioreactor were about 53% of the control (Figure 1) (Dept of Biochemical Eng. - UCL).

## Conclusions

We demonstrated that the iCELLis system could be very useful for production of viral vaccine and gene therapy vectors. The iCELLis platform facilitates handling and scale-up, high biomass amplification and sterile containment within a closed system. Moreover, in many cases, the specific culture environment enhances virus production yields.

Specifically, after some optimization of the culture parameters, it was demonstrated that rAAV vectors were produced by modified A549 cells in high viral level in the 0.53 m^2 ^iCELLis bioreactor. The maximum viral yield achieved in the bioreactor was 4.5 × 10^8 ^vg/cm^2^, which was higher than the yield per cm^2 ^obtained in a CellSTACK vessel (3.1 × 10^8 ^vg/cm^2^).

Finally, the preliminary results of transfection demonstrated that the method using PEI is applicable in the iCELLis bioreactors, with optimization of the viral recovery at harvest yet to be performed. This also demonstrated that the iCELLis can be considered as a solution for transient transfection processes at large scales.

